# Interaction Analysis of a Two-Component System Using Nanodiscs

**DOI:** 10.1371/journal.pone.0149187

**Published:** 2016-02-16

**Authors:** Patrick Hörnschemeyer, Viktoria Liss, Ralf Heermann, Kirsten Jung, Sabine Hunke

**Affiliations:** 1 Fachbereich Biologie/Chemie, Mikrobiologie, Universität Osnabrück, Barbarastrasse 11, D-49076, Osnabrück, Germany; 2 Munich Center for Integrated Protein Science (CiPSM) at the Department of Microbiology, Ludwig-Maximilians-Universität München, 82152, Martinsried, Germany; Oregon Health & Science University, UNITED STATES

## Abstract

Two-component systems are the major means by which bacteria couple adaptation to environmental changes. All utilize a phosphorylation cascade from a histidine kinase to a response regulator, and some also employ an accessory protein. The system-wide signaling fidelity of two-component systems is based on preferential binding between the signaling proteins. However, information on the interaction kinetics between membrane embedded histidine kinase and its partner proteins is lacking. Here, we report the first analysis of the interactions between the full-length membrane-bound histidine kinase CpxA, which was reconstituted in nanodiscs, and its cognate response regulator CpxR and accessory protein CpxP. Using surface plasmon resonance spectroscopy in combination with interaction map analysis, the affinity of membrane-embedded CpxA for CpxR was quantified, and found to increase by tenfold in the presence of ATP, suggesting that a considerable portion of phosphorylated CpxR might be stably associated with CpxA *in vivo*. Using microscale thermophoresis, the affinity between CpxA in nanodiscs and CpxP was determined to be substantially lower than that between CpxA and CpxR. Taken together, the quantitative interaction data extend our understanding of the signal transduction mechanism used by two-component systems.

## Introduction

Two-component systems are the predominant class of signal transduction systems that enable bacteria to cope with environmental change. The classical two-component system comprises a sensor histidine kinase and a response regulator [1]. Upon stimulation, the kinase is autophosphorylated at a conserved histidine residue. The phosphoryl group is subsequently transferred to a conserved aspartate in the cognate response regulator, which then mediates the response. The response is terminated either by “passive” dephosphorylation of the response regulator owing to intrinsic instability, or by the kinase activity itself in the case of bifunctional histidine kinases [1]. Importantly, a high degree of specificity between a histidine kinase and its cognate response regulator facilitates precise regulation and prevents unwanted cross-talk between unrelated two-component systems [2, 3]. This specificity is provided by specific amino acid residues on the interaction surface between the sensor histidine kinase and the response regulator, which determine the relative affinity between such signaling proteins [4–6]. The measured affinities (K_D_) between cognate signaling proteins range between 1 and 5 μM [4, 7–11].

Some two-component systems are additionally regulated by an accessory protein [12–14]. Such proteins can be found in all compartments of a bacterial cell. In Gram-negative bacteria such as *Escherichia coli* the cytosol is delimited by a peptidoglycan envelope, which is composed of an inner membrane, the periplasmic space (including the peptidoglycan layer) and an outer membrane. Furthermore, the envelope bears exposed surface structures such as flagella, secretion systems and adhesins [15]. Several cytosolic accessory proteins have been shown to control the metabolic status of the cell by modulating the interaction between sensor histidine kinase and response regulator, and thus the final readout of a two-component system [16]. But how most accessory proteins in the envelope influence signal transduction by two-component systems remains unclear, even in cases where the sensor histidine kinase activity targeted by them is known [17]. However, no quantitative data relating to the interaction between an accessory protein and its cognate kinase are currently available.

**Fig 1:**
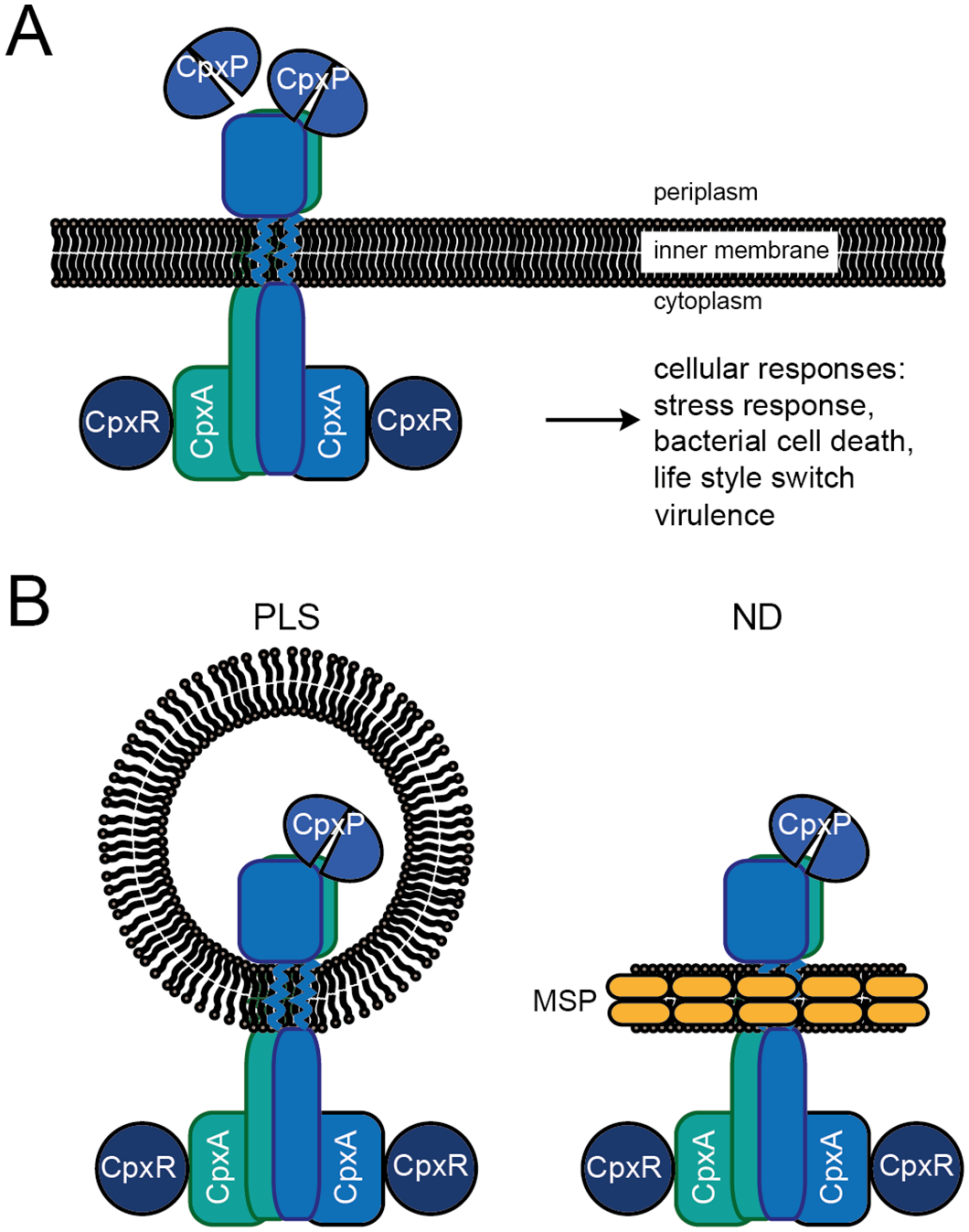
Schematic representation of the Cpx system and its incorporation into lipid bilayer systems for functional analysis. (A) The sensor histidine kinase CpxA is located in the inner membrane as a symmetrical homodimer. Upon stimulation, trans-autophosphorylation commences and phosphotransfer to the response regulator CpxR takes place [30]. Phosphorylation of CpxR activates a variety of responses. The accessory protein CpxP acts as a dimer to maintain CpxA in an inactive state [17, 31]. (B) Schematic representation of CpxA incorporated into a proteoliposome (left) or a nanodisc (right). By employing nanodiscs (ND), it is possible to avoid certain experimental limitations associated with proteoliposomes (PLS), e.g. limited accessibility of the target protein and their large und unstable nature. NDs provide an essentially native and fully controllable nanoscale phospholipid bilayer and permit structural-functional experimentation in solution. The ND is composed of phospholipids that associate as a bilayer, which is bounded a ring formed by two amphiphilic Membrane-Scaffolding-Protein (MSP) molecules [32].

The Cpx two-component system (Cpx-TCS) of *E*. *coli* is an excellent model for investigations of signal integration, signal transduction and partner specificity in two-component signaling. The Cpx-TCS detects and responds to envelope perturbations by regulating the expression of chaperones and proteases, macromolecular surface structures and other stress-response systems [18–21]. Importantly, in pathogenic strains, the Cpx system has an impact on host colonization and biofilm formation [reviewed in [22–24]]. Thus the Cpx-TCS regulates the expression of key virulence transcriptional regulators, such as VirF of *S*. *flexneri* [25, 26], of virulence determinants such as the P pili of uropathogenic *E*. *coli* [27] and of colonization factors in *Xenorhabdus nematophila*. The Cpx-TCS consists of the sensor histidine kinase CpxA, the response regulator CpxR and the accessory protein CpxP (Fig 1A) [28, 29].

CpxA is a structural and functional homodimer and exhibits the typical architecture of sensor histidine kinases, including an extracytoplasmic sensor domain that contains a PAS fold and protrudes into the periplasm, two membrane-spanning transmembrane domains in the cytoplasmic membrane, and the cytoplasmic “effector moiety” [30, 33]. This last is linked to one of the transmembrane domains and is composed of a HAMP domain that comprises two helices. These encompass the DHp domain and form a four-helix bundle in the homodimer. The DHp domain in turn is connected via a loop-region to the catalytically active ATP-binding domain [29]. CpxR is a typical response regulator with an N-terminal receiver domain, which dimerizes upon phosphorylation by CpxA. Phosphorylation of CpxR activates the attached C-terminal output domain, which in turn effects regulation of gene expression. CpxP is a periplasmic protein that inhibits the Cpx system in a negative feedback loop [34]. The *cpxP* gene is the most strongly induced CpxR target gene under conditions of envelope stress [35]. Deletion of *cpxP* results in mild induction of the Cpx response, which can be further enhanced by other stimuli [34]. Interestingly, complete inhibition of the Cpx response by CpxP is achieved only when the latter is overproduced [34]. In addition to its inhibitory function, CpxP, together with the periplasmic protease DegP, is indispensable for quality control of misfolded envelope proteins that are misrouted during biogenesis [27, 35, 36]. Recent studies have demonstrated that the inhibitory and quality-control functions of CpxP are linked. CpxP acts as a homodimer that inhibits CpxA kinase activity through direct interaction in unstressed cells [17, 31, 37]. Salt stress and misfolded envelope proteins disrupt this interaction, thus inducing the Cpx response [37]. This dynamic interaction between the sensor histidine kinase CpxA and the accessory protein CpxP seems to be important for the function of the system [36]. Hence, it is of interest to quantitatively investigate the kinetics of this interaction. To date, the interaction has been investigated on a functional basis using proteoliposomes (PLS), at the structural level using peptide arrays or qualitatively using mSPINE [17, 31, 37]. Accordingly, quantitative parameters of the interaction are lacking and should preferably be determined by independently monitoring association (k_a_) and dissociation (k_d_) rates. These kinetic parameters of interaction can then be correlated with the physiological state of the cell.

Here, we have investigated the affinities of the reconstituted sensor histidine kinase CpxA for its cognate response regulator CpxR and its accessory protein CpxP in order to understand the functionality and dynamics of the system. We chose to reconstitute CpxA into nanodiscs (ND), because this would allow us to carry out affinity measurements with partner proteins from both sides of the membrane. NDs are composed of a membrane protein, a nanoscale phospholipid bilayer and two copies of a genetically modified “Membrane Scaffolding Protein” (MSP), which wrap around the disc-like bilayer (Fig 1B, right panel) [38]. Our results demonstrate that the CpxA-ND has a high affinity for its cognate response regulator CpxR, which is increased approximately 10-fold upon phosphorylation. Importantly, the ND approach allowed us for the first time to determine the affinity between a membrane-embedded sensor histidine kinase and its accessory protein. Interestingly, the affinity between CpxA-ND and the accessory protein CpxP is much lower than that between CpxA and CpxR.

## Materials and Methods

### Overproduction and purification of MSP1D1

The sequence encoding the membrane scaffolding protein MSP1D1 was subcloned from pMSP1D1 (Addgene) into pBAD24 using NcoI and HindIII restriction sites. MSP1D1 was produced in *E*. *coli* TG1 cells grown in TB medium (per liter: 12 g tryptone, 24 g yeast extract, 5 g glycerol; 10x stock TB salts per liter: 23.1 g KH_2_PO_4_, 125.4 g K_2_HPO_4_), purified as described in [32], aliquoted and stored at -80°C. For SPR measurements, the attached His-tag was removed by treatment with TEV protease. The TEV protease, cleaved His-tags and uncleaved MSP1D1 were removed by adding Ni-NTA Agarose (Invitrogen), stirring for 1 h at 4°C and pelleting the resin using Pierce^®^ Centrifuge Columns, as verified by immunoblot analysis with antibodies directed against the His-tag.

### Overproduction and purification of *E*. *coli* CpxA, CpxR and CpxP

CpxA-His6, His6-CpxR and His6-CpxP were overproduced in *E*. *coli* BL21(DE3) <pLysS> (Novagen) from pI3CpxA, pI4CpxR, and pRF6, respectively, and purified as described previously [17]. CpxA-Strep was overproduced in the same host from pI1CpxA. Cells were grown at 30°C in a 5-l fermenter (Biostat B, Sartorius) and harvested 3 h after induction of *cpxA* expression with 0.5 mM IPTG. Membrane vesicles were prepared as described in [37], and solubilized in 50 mM HEPES (pH 7.5), 300 mM NaCl, 300 mM KCl and 1% (w/v) *n*-dodecyl β-D-maltoside (DDM). The protein was purified by using 1-ml Strep-Tactin^®^ Superflow^®^ columns (IBA GmbH) according to the manufacturer’s instructions. The major fractions were pooled and concentrated, and the buffer was changed to 50 mM HEPES (pH 7.5), 100 mM NaCl, 100 mM KCl and 0.1% DDM. Proteins were then aliquoted, shock-frozen in liquid nitrogen and stored at -80°C.

### ND and PLS assembly

To promote formation of nanodiscs (ND) in accordance with Denisov et al. [32], a molar ratio of protein-to-lipid of 1:65 was chosen using “*E*. *coli* Extract Total” by Avanti^®^. MSP-to-CpxA molar ratios were generally set to 1:1. Proteoliposomes were created according to [17], except that β-cyclodextrin was used instead of polystyrene beads to remove detergent, as described in the following. The reconstitution mixtures were incubated at room temperature (RT) for 5 min. Reconstitution was carried out using β-cyclodextrin essentially as described in [39]. Thus PLS or ND formation was initiated by adding a 2-fold excess of β-cyclodextrin over detergent (DDM) and mixing rapidly. Samples were centrifuged for 5 min at 16,000g to remove aggregates prior to further experimentation.

### Analytical size-exclusion chromatography (SEC)

To monitor successful ND formation, analytical SEC was conducted on a Superdex^™^ 200 10/300 GL column at RT as described in [32]. The flow rate was 0.3 ml/min and the buffer consisted of HEPES-buffered saline (20 mM HEPES (pH 7.5), 150 mM NaCl). Control experiments were conducted by applying MSP monomers (40 μM) in 500 μl buffer, or empty ND (40 μM MSP in 500 μl). For the CpxA-ND run, 500 μl of reconstituted sample containing MSP (40 μM) and CpxA (27 μM) was applied and peaks were subsequently pooled, concentrated via Amicon Ultra 0.5 ml Centrifugal Filters (Ultracel -10K), and analyzed by 10% SDS-PAGE. Fractions were analyzed for phosphotransfer activity as described in Supplemental Materials.

### TEM

For negative staining of proteins Formvar-coated 400-mesh copper grids (Plano) were used. CpxA in ND or PLS was diluted in buffer (50 mM Tris/HCl pH 7.5, 150 NaCl) to a concentration of 1 μM, and a drop of the solution was applied to a grid and incubated for 1 min. After removing excess protein solution with filter paper, the grid was washed thrice with H_2_O and immediately stained with filtered 2% (w/v) uranyl acetate for 1 min. Excess stain was removed with filter paper and the grid was dried for at least 1 h at RT in the dark. TEM was performed using a Zeiss EM 902A microscope operated at 50 kV and equipped with 2K wide-angle slow-scan CCD camera (TRS). Images were taken at 250,000x magnification and analyzed with ImageSP software (TRS image SysProg).

### Autophosphorylation assay

Autophosphorylation activity was determined according to (16). In brief, CpxA-His6 was reconstituted in PLS or ND at a final concentration of 1.5 μM. The reaction mixture consisted of 50 mM KCl and Tris-glycerol-DTT buffer (50 mM Tris/HCl pH7.5, 10% glycerol (v/v), 2 mM dithiothreitol). The kinase reaction was initiated by adding the appropriate volume of a 20x ATP-Mix (100 mM MgCl_2_, 0.9 mM ATP, 30 μCi [γ-^32^P]ATP, and Tris-glycerol-DTT buffer). Samples were taken after 1, 5, 10, 20 and 30 min, and the reaction was stopped by addition of 5x SDS sample buffer [40]. Samples were immediately subjected to SDS-PAGE and after the run the gels were dried for 3 h under vacuum and heating at 70°C. The gels were then exposed to phosphorimager plates overnight and analyzed using a STORM^™^ 820 phosphorimager and Quantity One^®^ software.

### Phosphotransfer assay

CpxA-His6 was reconstituted at a final concentration of 5 μM in PLS or ND, and His6-CpxR was added (final concentration 5 μM). The reaction was started by adding the appropriate volume of a 20x ATP-Mix (100 mM MgCl_2_, 0.9 mM ATP, and Tris-glycerol-DTT buffer). Samples were taken after 1, 5, 10, and 15 min, the reaction was stopped by addition of 5x SDS sample buffer, and then subjected to analysis by Zn^2+^-Phos-tag^™^ PAGE (50 μM Phos-tag acrylamide, 10% (w/v) acrylamide) as described [41]. As a control, 5 μM CpxR was chemically phosphorylated by acetyl-phosphate, using 5 μM CpxR, 10 mM acetyl-phosphate in phosphorylation buffer (50 mM Tris/HCl pH 7.6, 50 mM KCl, 20 mM MgCl_2_) and Tris-glycerol-DTT buffer for 20 min as described. Gels were stained with Coomassie blue, scanned and analyzed using GelQuantNet software (BiochemLabSolutions.com).

### Surface plasmon resonance assays

SPR assays were performed in a Biacore T200 using Xantec CMD500-D carboxymethyl dextran sensor chips (XanTec Bioanalytics GmbH) that had been coated with anti-His antibodies from the Biacore His-capture kit (GE Healthcare), as this chip surface allows for complete regeneration of His-tagged molecules from a sensor chip in a capturing SPR approach. The chips were first equilibrated with HBS-EP buffer (10 mM HEPES pH 7.4, 150 mM NaCl, 3 mM EDTA, 0.005% (v/v) detergent P20) until the dextran matrix had swollen. Then, two of the four flow cells on each sensor chip were activated by injecting a 1:1 mixture of N-ethyl-N-(3-dimethylaminopropyl)carbodiimide hydrochloride and N-hydroxysuccinimide using the standard amine-coupling protocol. Both flow cells were then loaded with a final concentration of 50 μg/ml of anti-His antibody in 10 mM acetate (pH 4.5) using a contact time of 420 s, so that the surfaces contained antibody densities equivalent to ~10,000 resonance units (RU). Free binding sites on the flow cells were saturated by injection of 1 M ethanolamine/HCl (pH 8.0). Preparation of chip surfaces was carried out at a flow rate of 10 μl/min. Analyses of interaction between CpxR-His6 and CpxA in nanodiscs (CpxA-ND) were then performed in HBS buffer (10 mM HEPES (pH 7.4), 150 mM NaCl). First, CpxR-His6 (50 nM) was captured in the second flow cell using a contact time of 60 s at a constant flow rate of 10 μl/min, followed by a stabilization time of 20 s so that approximately 300 RU of CpxR-His6 were captured. Increasing concentrations (25 nM, 50 nM, 2x 100 nM, 250 nM, 500 nM and 1000 nM) of CpxA-ND or phosphorylated CpxA-P-ND (in the presence of 2 mM Mg^2+^-ATP) were then injected onto both flow cells using a contact time of 180 s each and a final dissociation step (600 s) using a flow rate of 30 μl/min. As a control, similar concentrations of empty nanodiscs were injected onto the chip. After each cycle the chip was regenerated by injection of 10 mM glycine (pH 1.5) for 60 s at a flow rate of 30 μl/min over both flow cells, which completely removes CpxR-His6 from the surface. All experiments were performed at 25°C. Sensorgrams were recorded using Biacore T200 Control software 1.0 and analyzed with Biacore T200 Evaluation software 1.0. The surface of flow cell 1 was used to obtain blank sensorgrams for subtraction of the bulk refractive index background. The referenced sensorgrams were then normalized to a baseline of 0. Spikes in the sensorgrams at the start and the end of the injections emerged from the run-time difference between the flow cells on each chip.

### Interaction Map^®^ (IM) analysis

IM calculations were performed on the Ridgeview Diagnostic Server (Ridgeview Diagnostics). For this purpose, the SPR sensorgrams were exported from the Biacore T200 Evaluation Software 1.0 as *.txt files and imported into the TraceDrawer Software 1.5 (Ridgeview Instruments, Uppsala, Sweden). IM files were created using the IM tool included in the software, generating files that were sent via e-mail to the server (im@ridgeviewdiagnostics.com) where the IM calculations were performed [42]. The resulting files where then evaluated for spots in the TraceDrawer 1.5 software, and the IM spots were quantified.

### Microscale thermophoresis

Affinity measurements using microscale thermophoresis (MST) were carried out with a Monolith NT.115 instrument (NanoTemper Technologies). CpxA-Strep or MSP1D1 were labeled using the NanoTemper NHS NT-647 labeling kit. The labeling reaction was performed according to the manufacturer’s instructions, and applying a final concentration of 20 μM protein with a 3x molar excess of dye at RT for 30 min in the dark. Free dye was eliminated using the supplied dye-removal columns equilibrated with CpxA-Strep storage buffer (50 mM HEPES (pH 7.5), 100 mM NaCl, 100 mM KCl, 0.1% DDM), or HEPES-buffered saline (20 mM HEPES (pH 7.5), 150 mM NaCl), respectively. Labeled CpxA-Strep was reconstituted into nanodiscs at a final concentration of 300 nM as described above. CpxR or CpxP was diluted in HEPES-buffered saline creating a dilution series of 16 1:1 dilutions (735 to 0.02 μM for CpxP and 475 to 0.014 μM for CpxR). For the thermophoresis experiment, each ligand dilution was mixed 1:1 with 300 nM labeled CpxA-Strep in nanodiscs, yielding a final CpxA concentration of 150 nM and a dilution series of 367.5 to 0.01 μM for CpxP and 237.5 to 0.007 μM for CpxR. To analyze the effect of ATP on the affinity between CpxA and CpxR, the CpxA-Strep nanodiscs were supplemented with 500 μM ATP and 5 mM MgCl_2_ before mixing with CpxR dilutions. All experiments were incubated for 2 min at RT, before applying samples to Monolith NT Standard Treated Capillaries (NanoTemper Technologies). Thermophoresis was measured at RT with 5 s/30 s/5 s laser off/on/off times. Experiments were conducted at 50% LED power and 20% MST IR-laser power. Data from at least three independently performed experiments were analyzed (NT.Analysis software version 1.5.41, NanoTemper Technologies) using the signal from Thermophoresis + T-Jump. Baseline correction was performed and data were averaged and fitted using the law of mass action.

## Results

### Reconstitution of CpxA into nanodiscs

In order to improve our knowledge of the functionality and dynamics of the Cpx system, we set out to measure the affinities of the sensor histidine kinase CpxA for its cognate response regulator CpxR and its periplasmic accessory protein CpxP. In particular, we wished to determine for the first time the interaction kinetics between a sensor histidine kinase (CpxA) reconstituted in a lipid bilayer and partner proteins that interact with it from different sides of the membrane (CpxR is a cytosolic protein, whereas CpxP is found in the periplasm). This precluded the use of the well-established liposome-based *in vitro* signal transduction system [17], as CpxA is incorporated into proteoliposomes (CpxA-PLS) in inverse orientation (Fig 1B, left panel), rendering the periplasmic sensor domain of CpxA inaccessible to externally added CpxP. Moreover, we could not use detergent-solubilized CpxA for affinity measurements, since detergent-solubilized CpxA and CpxA-PLS differ in their biochemical properties, as previously demonstrated by inhibitor studies [17]. Consequently, we chose to reconstitute CpxA in nanodiscs (CpxA-ND) (Fig 1B, right panel), which allowed us to investigate CpxA signaling in a native lipid bilayer affording full accessibility to all moieties of the protein. Membrane-protein reconstitution is conventionally achieved by employing appropriate molar ratios of MSP to transmembrane protein to phospholipid and then removing the detergent [38]. Here, we used instant detergent removal mediated by β-cyclodextrin instead of the polystyrene-bead approach to initiate reconstitution of CpxA [39].

**Fig 2:**
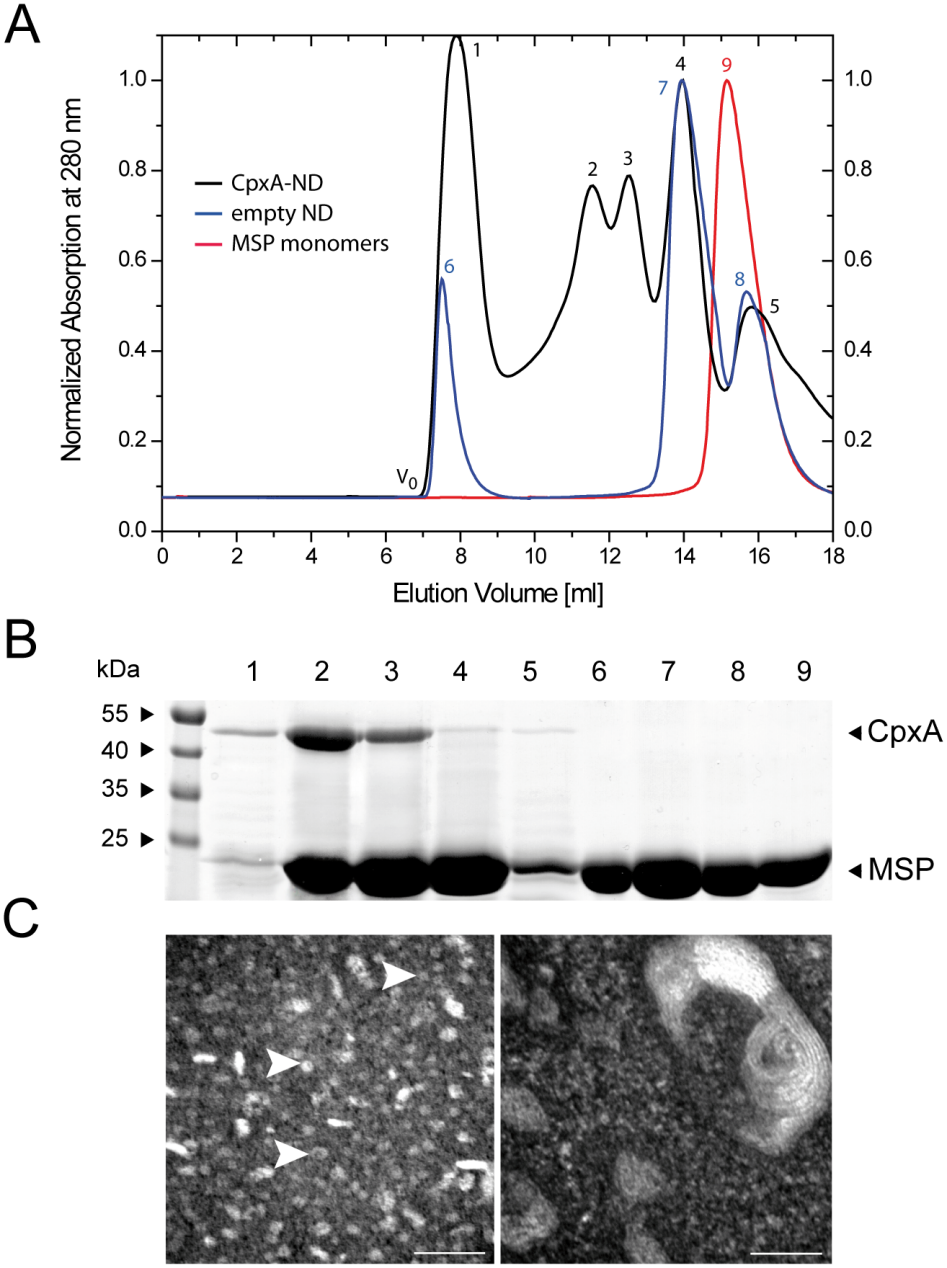
Characterization of nanodiscs containing sensor histidine kinase CpxA (CpxA-ND). (A) Analytical size-exclusion chromatography. Elution profiles of the pure MSP (MSP monomers, red), empty nanodiscs (blue), and CpxA-ND-complexes (black), respectively. Peaks are numbered (see text for details). (B) SDS-PAGE analysis after sample concentration using Amicon Ultra Centrifugal Filters of the peaks labeled in *A*. (C) TEM analysis of CpxA-NDs (left) and CpxA-PLS (right). The scale bar represents 50 nm. CpxA-ND samples contain characteristic ring-like structures (indicated by white arrows) of about 10 nm in diameter. The CpxA-PLS (right image) shows multilaminar structures with diameters of 50 nm in size and more.

Reconstitution of the sensor histidine kinase CpxA into ND was monitored by analytical size-exclusion chromatography (SEC) (Fig 2A) [32]. A sample of purified membrane scaffolding protein (MSP1D1) served as a control and as a marker for the elution volume of unassembled NDs, i.e. MSP monomers (Fig 2A, red curve, peak 9). The peak was analyzed by SDS-PAGE and only MSP1D1 (24.7 kDa) was detected (Fig 2B, lane 9). As a second control, general ND assembly was also examined (Fig 2A, empty ND, blue curve) using a molar ratio of 1:65 of MSP1D1 to lipids, in accordance with Denisov *et al*. [32]. The distinctive shift of the curve (peak 7) to lower elution volumes demonstrates that the use of cyclodextrin for detergent extraction permits successful assembly of NDs. The first detected peak (Fig 2A, blue curve, peak 6) eluted at the void volume (V_0_) and presumably represents aggregated NDs. Only MSP1D1 could be detected (Fig 2B, lane 6). Analysis of the putative ND peak at 14 ml (Fig 2A, blue curve, peak 7; Fig 2B, lane 7) and the consecutive peak (blue curve, peak 8; Fi 2B, lane 8) revealed only MSP1D1 (Fig 2B), with the consecutive peak representing monomeric MSP1D1 [32]. Injection of the reconstitution mixture consisting of ND with CpxA produced five discernible elution peaks (Fig 2A, CpxA-ND, black curve). Peak 1 was obtained at V_0_ and contained presumably aggregated CpxA and MSP1D1, as shown in lane 1 (Fig 2B). Peaks 2 and 3 correspond to species of NDs containing different copy numbers of CpxA per ND. Based on the SDS-PAGE patterns, where lane 2 shows a significantly more intense band at around 52 kDa than does lane 3 (Fig 2B), together with the fact that CpxA forms a stable dimer [30], it is conceivable that peak 3 contains one CpxA dimer and peak 2 two CpxA dimers per ND. Peak 4 eluted at the same elution volume as empty NDs (peak 7) and was confirmed by SDS PAGE to contain mostly MSP1D1 (Fig 2B, lane 4), whereas peak 5 eluted at the elution volume of monomeric MSP1D1 (compare with peaks 8 and 9). Analysis confirmed the prevalence of MSP1D1 in peak 5 (Fig 2B, lane 5). Moreover, the functionality of possible CpxA contents in peaks 1–5 was investigated to further elucidate the efficacy of CpxA reconstitution (S1 Fig).

Next, we investigated whether or not CpxA-ND specimen contained any PLS as side products by subjecting samples of CpxA-ND or CpxA-PLS to transmission electron microscopy (TEM) (Fig 2C). In the CpxA-ND sample, we found structures that corresponded in size to the theoretical ND diameter of 10 nm (Fig 2C, left image) [32]. The rod-like structures that can also be seen (Fig 2C, left image) presumably represent CpxA-ND complexes that are flipped onto their sides due to the loading with membrane protein (CpxA), as suggested by Shi *et al*. for SNARE complexes in ND [43]. The TEM image on the right in Fig 2C shows CpxA-PLS. These liposome structures were 50 nm in size, and clearly larger than the structures observed in the CpxA-ND sample. Additionally, multilaminar structures were observed, which resemble TEM images of liposomes obtained by Gobin *et al*. [44] and DeGrip *et al*. [39].

### CpxA embedded in nanodiscs is active

**Fig 3:**
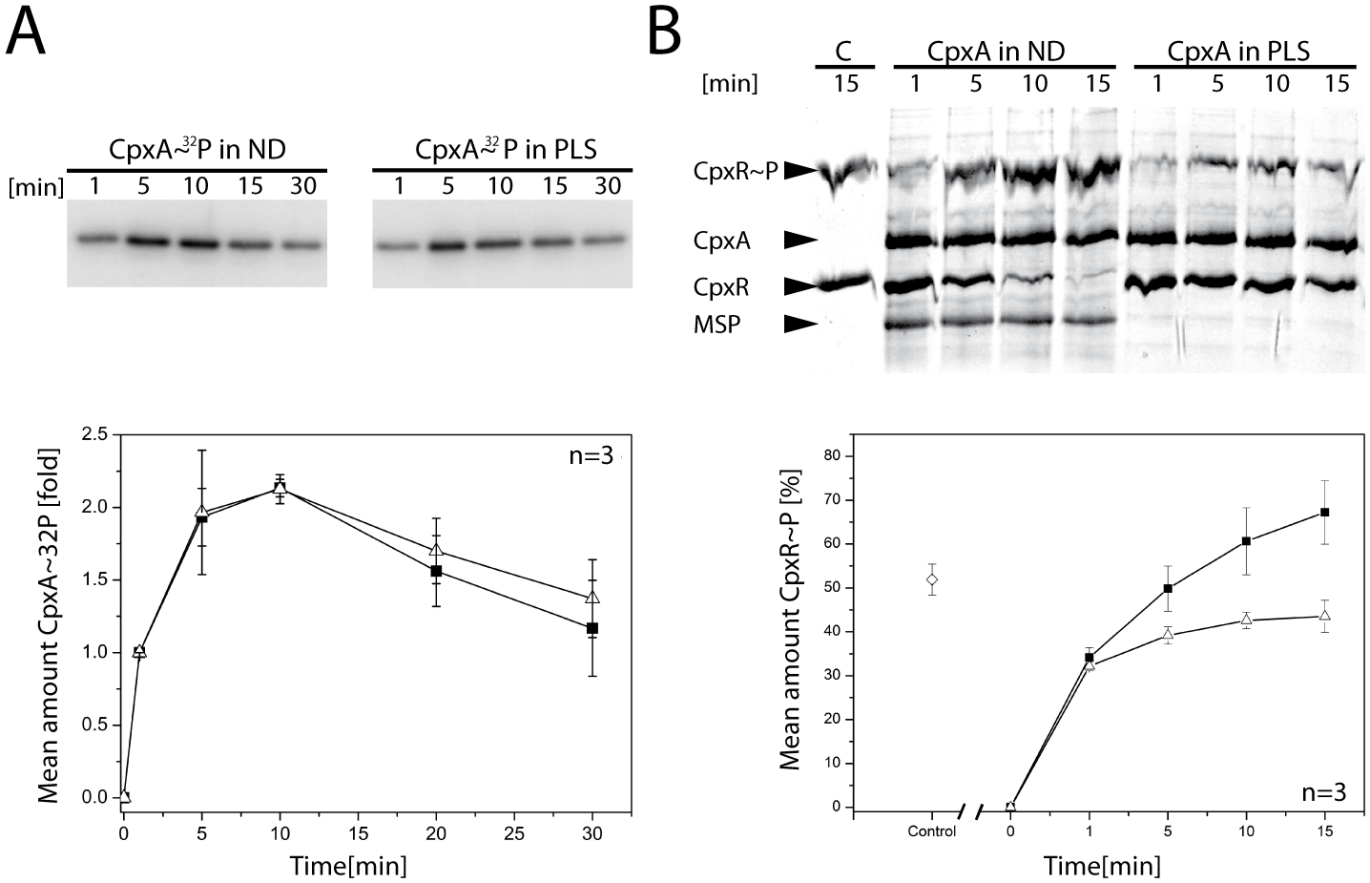
CpxA is functional in nanodiscs. (A) Phosphorylation activities of the sensor histidine kinase CpxA in ND and PLS. Autophosphorylation was initiated by addition of [γ^32^-P]ATP. Samples were withdrawn at the indicated time points. The plot (lower panel) shows the results of densitometric analysis (of triplicate gels) of the autoradiograms in (A) The amount of CpxA~^32^P seen after 1 min was set to 1 and used for normalization of the other values. (B) Phosphotransfer from CpxA to CpxR. CpxR was phosphorylated using CpxA in ND (■) and in PLS (ρ), respectively, and a representative gel is shown (the experiment was performed in triplicate). A control experiment (lane marked with C; ◇ in the plot) was carried out by phosphorylating CpxR with acetyl-phosphate for 15 min. Phosphotransfer was initiated by concomitant addition of CpxR and ATP. Samples were withdrawn at the indicated time points and subjected to a Zn^2+^-Phos-tag^™^ PAGE. Densitometric analysis of the CpxR~P bands was performed and mean values were plotted (lower panel). Error bars indicate the standard deviation.

In order to examine whether the sensor histidine kinase CpxA is functional in ND, we compared the biochemical activities of full-length CpxA incorporated into either ND or PLS. First, we investigated the autokinase activity of CpxA by addition of [γ-^32^P]ATP. The activity was assayed for 30 min in total (Fig 3A).

Samples were withdrawn at the indicated time points, and the reaction was quenched by addition of SDS sample buffer. The samples taken at the first time point (i.e. 1 min) were set as the initial quantity of phosphorylated CpxA. In both experimental approaches, the amount of CpxA~^32^P increased more than twofold within the first 10 min. The autophosphorylation rate, as well as the maximal phosphorylation of CpxA, was similar for ND and PLS (Fig 3A). Amounts of CpxA~^32^P decreased after 10 min, an effect which has also been observed for the histidine kinase ArcB [45].

Next, the efficacy of phosphotransfer activity was assayed for CpxA-ND and CpxA-PLS using Phos-tag^™^ PAGE [46]. The readout using Phos-tag^™^ PAGE was sufficiently clear-cut to make radiolabeling unnecessary (Fig 3B). CpxA was reconstituted as described before, incubated with CpxR in the presence of ATP, and samples were withdrawn after 1, 5, 10 and 15 min. As control, CpxR was chemically phosphorylated with acetyl-phosphate, which is capable of phosphorylating response regulators [47, 48], as donor. The ratio of phosphorylated to non-phosphorylated CpxR was determined by densitometry after SDS-PAGE (Fig 3B). Interestingly, the analysis clearly showed an increase in phosphotransfer rate and a higher final level of CpxR~P for CpxA-ND compared to CpxA-PLS (Fig 3B), with amounts of CpxR~P after 15 min being approximately 1.5-fold higher in CpxA-ND than in CpxA-PLS samples. Apparent saturation (after 15 min) at around 40% CpxR~P was achieved for CpxA-PLS, whereas the level observed with ND was about 65% CpxR~P (Fig 3B), indicating that CpxA in ND retains more phosphotransfer activity. Together, these results demonstrate that the sensor histidine kinase CpxA is functional in ND.

### ATP potentiates the interaction between CpxA and CpxR

**Fig 4:**
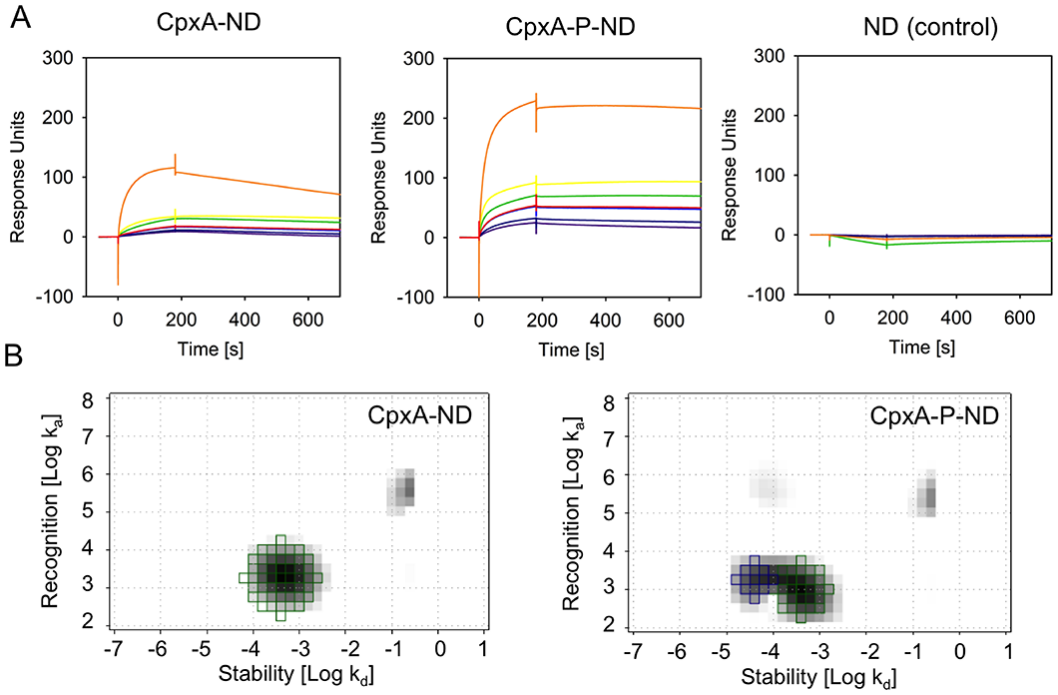
Binding of CpxA-ND and CpxA-P-ND to CpxR. (A) Binding of CpxA-ND to CpxR-His6 was analyzed by SPR. CpxR-His6 was captured via the His-tag onto a CM5 sensor chip coated with anti-His antibody, and solutions of 25 nM (*purple*), 50 nM (*dark blue*), 100 nM (*blue* and *red*), 250 nM (*green*), 500 nM (*yellow*), 1000 nM (*orange*) of CpxA-ND, CpxA-P-ND or equal amounts of empty ND were passed over the chip. The data presented come from one of three independently performed experiments. (B) Interaction Maps^®^. The green squares represent the CpxA-ND/CpxR interaction (k_a_ = 2.01 x 10^3^/M*s; k_d_ = 4.73 x 10^−4^/s; K_D_ = 236 nM), the blue squares the CpxA-P-ND/CpxR interaction (k_a_ = 1.76 x 10^3^/M*s; k_d_ = 4.38 x 10^−5^/s; K_D_ = 24.8 nM).

After establishing functional reconstitution of the sensor histidine kinase CpxA into ND we sought to investigate the kinetic parameters of the interaction between CpxA-ND and its cognate response regulator CpxR. We used SPR to determine the kinetics (ON/OFF rates) of binding of CpxA in real time. To this end, His6-CpxR was captured onto a sensor chip that had been preloaded with anti-His antibodies. Then, increasing concentrations of CpxA-ND (25 nM-1000 nM) were injected. After each cycle, His6-CpxR was regenerated from the chip and recaptured. We clearly detected increased binding of CpxA-ND to CpxR with increasing CpxA-ND concentrations (Fig 4A, left panel).

Furthermore, when CpxA-ND was first phosphorylated (CpxA-P-ND) by adding ATP and MgCl_2_, subsequent binding of CpxR was enhanced, as revealed by an approximately two-fold increase in the maximal binding rate (Response Units; see Fig 4A, middle panel). Moreover, dissociation rates were clearly decreased when CpxA-P-ND were used instead of CpxA-ND, indicating a stabilization of the complex. As expected, ND without CpxA showed no binding to His6-CpxR, confirming the specificity of the sensorgrams (Fig 4A, right panel). However, the sensorgram shapes did not conform to the typical 1:1 binding curve, as those obtained with high concentrations of CpxA-ND or CpxA-P-ND did not reach saturation, indicating that the curves record a combination of different binding events. This multivalent interaction might be caused by non-specific interaction of the NDs with each other at high concentrations. To quantify the binding kinetics, we calculated Interaction Maps^®^ (IM) [42]. Briefly, the IM algorithm splits the experimental SPR dataset into several theoretical monovalent binding curves and identifies the binding curves which together best fit the experimental data. By plotting the association rate k_a_ and the dissociation rate k_d_ within a two-dimensional distribution it is then possible to display heterogeneous binding data as a map in which each peak corresponds to one component that contributes to the cumulative binding curve. As shown in Fig 4B, the IM calculated for the CpxA-ND-CpxR interaction shows one major peak with an association rate k_a_ of 2.01 x 10^3^/M*s and a dissociation rate k_d_ of 4.73 x 10^−4^/s. The overall binding affinity (K_D_) for this interaction would therefore be at 236 nM. Furthermore, a small peak with a high association and high dissociation rate appeared but, since the weight of this peak was calculated to be less than 10%, it was assumed to reflect unspecific binding of the NDs to each other or a bulk effect, and therefore neglected. The IM calculated for the CpxA-P-ND-CpxR interaction resulted in two major peaks, one representing the interaction of non-phosphorylated the sensor histidine kinase CpxA with the response regulator CpxR. Furthermore, a peak appeared representing a 10-fold higher affinity (K_D_ = 24.8 nM), which we interpret as the interaction of CpxA-P-ND with CpxR. The association rate for this interaction is comparable to that found for non-phosphorylated CpxA (k_a_ = 1.76 x 10^3^/M*s), and the lower dissociation rate (k_d_ = 4.38 x 10^−5^/s) accounts for the higher affinity, as was already discernible in the sensorgrams. The two peaks with high association rates also had peak weights below 10% and were therefore not taken into account.

To the best of our knowledge, this study quantifies for the first time the interaction between a full-length sensor histidine kinase and its cognate response regulator in their native, membrane-bound, state. Our data clearly show that ATP increases the affinity between the sensor histidine kinase CpxA and its cognate response regulator CpxR, and that the enhanced affinity is mainly caused by decreased dissociation rather than increased association rates.

### CpxP displays only weak affinity for CpxA

Next, we used CpxA-ND to measure the interaction between a sensor histidine kinase and its accessory protein. However, attempts to measure the affinity between CpxA-ND and its accessory protein CpxP using surface-sensitive detection techniques failed to yield reliable results (data not shown), indicating that the two proteins have very weak affinity for each other or the covalent coupling might interfere the binding event. Hence, we resorted to microscale thermophoresis (MST) to quantify the strength of interaction between CpxA-ND and its periplasmic accessory protein CpxP. For that purpose, CpxA was fluorescently labeled using the NHS NT-647 labeling kit, which is based on the idea that typically one primary amine per protein is labelled following a statistical distribution of the labelling position, and that the most molecules are not labelled in the vicinity of the interaction site [49, 50]. Labeled CpxA was then reconstituted in a 1:1 molar ratio into ND and the final concentration was kept constant at 150 nM.

**Fig 5:**
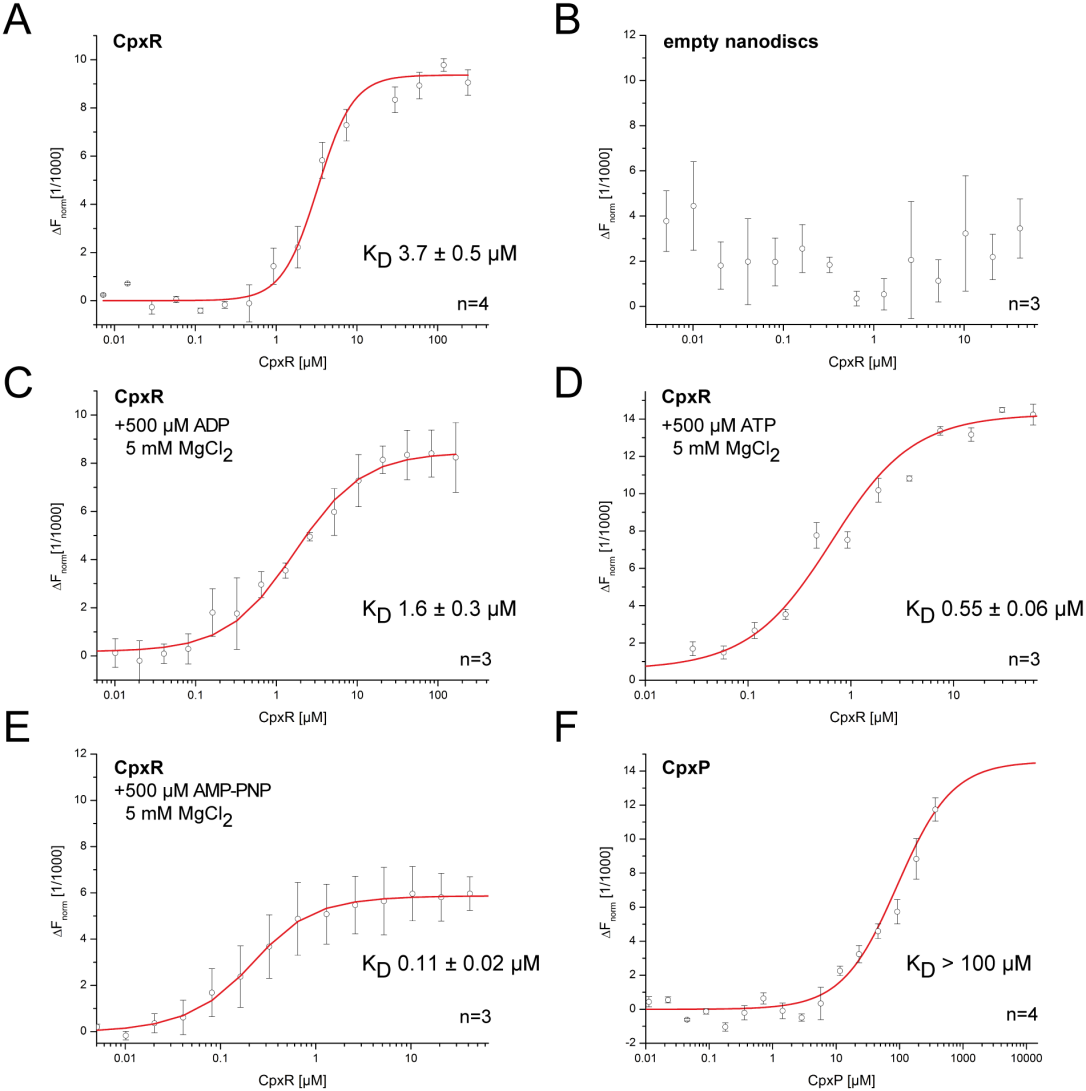
Affinities of CpxA-ND to CpxR in presence of nucleotides as well as CpxA-ND to CpxP. Affinities were measured using MST (A) Interaction between CpxA and CpxR. (B) Negative control: Empty NDs were titrated with CpxR. (C) Interaction between CpxA-ND and CpxR in presence of ADP+MgCl_2_ to mimic the phosphatase-competent state. (D) Interaction between CpxA and CpxR in the presence of ATP and MgCl_2_. (E) Interaction between CpxA-ND and CpxR in presence of AMP-PNP+MgCl_2_ to trap CpxA in the state prior to phosphotransfer. (F) Interaction between CpxA and CpxP. The concentration of the NT-647 labeled CpxA-ND was kept constant at 150 nM, and the concentrations of the non-labeled binding partners were varied (0.01 to 367.5 μM for CpxP and 0.007 to 237.5 μM for CpxR). (n = number of independent measurements; error bars indicate the standard deviation).

First we quantified the interaction of CpxA-ND with its cognate response regulator CpxR as a proof of principle and compared the affinity to that determined with SPR technique. For that purpose, CpxR was diluted in a 16-step 1:1 dilution series and mixed 1:1 with CpxA-ND solution, resulting in final ligand concentrations of 237.5–0.007 μM for CpxR (Fig 5A). Additionally, control experiments with empty NDs (fluorescent labeled MSP1D1, 150 nM final concentration) were conducted, showing no interaction between NDs and CpxR (Fig 5F). The K_D_ between CpxA-ND and CpxR was determined to be 3.7±0.5 μM (Fig 5A), 10-fold higher than the value (236 nM) determined with SPR. One possible reason for this difference is that the MST binding curve too represents a mixture of binding events and, unlike SPR, MST is limited to providing thermodynamic affinity constants at steady state and does not derive them from association and dissociation rates. Also, the interaction might be perturbed by the NHS ester fluorescent label tethered to CpxR. In the next experiment the phosphatase-competent state of CpxA was mimicked by addition of ADP and MgCl_2_ (Fig 5C). The resulting affinity of 1.6 ± 0.3 μM was in the same order of magnitude as for the experiment in absence of nucleotides (Fig 5A). In addition, one series of experiments was designed to monitor the effect of phosphorylation on the CpxA-CpxR interaction (Fig 5D). With MST we also observed a 10-fold increase in the affinity (0.55±0.06 μM) of CpxA-ND for CpxR in the presence of ATP, similar to that determined by SPR. The nucleotide-dependent effect was further confirmed by addition of the non-hydrolysable ATP-analogue AMP-PNP (Fig 5E), which mimics the nucleotide-bound state before ATP hydrolysis. A K_D_ of 0.11 ± 0.02 μM was observed which depicts a five-fold higher affinity for CpxA-ND to CpxR compared to the presence of ATP, but still remains in the same range. This result could be ascribed to the fact that rapid ATP hydrolysis gives rise to a heterogeneous mixture of kinase-competent and phosphatase-competent states of CpxA, whereas AMP-PNP solely favors the kinase-competent state.

Overall, these experiments clearly support the idea that MST is suitable for quantification of the interaction between CpxA-ND and its cognate response regulator CpxR. Hence, MST was also employed to determine the affinity between CpxA-ND and its accessory protein CpxP. CpxP was diluted in a 16-step 1:1 dilution series and mixed 1:1 with CpxA-ND resulting in final ligand concentrations of 367.5–0.01 μM CpxP. Although we employed the maximum stock concentration of CpxP, which is close to the critical concentration for precipitation, we could not distinctively obtain the point of saturated binding of CpxP to CpxA-ND. Nevertheless, we were able to estimate the K_D_ to be > 100 μM, which confirms the relative lack of affinity between both proteins (Fig 5E). In summary, the combination of ND and MST has allowed us to quantify the affinity between a histidine kinase and its periplasmic accessory protein.

## Discussion

The stability of the signaling complex is crucial for the efficacy of a stress-sensing system, such as the Cpx envelope-stress two-component system. Additionally, accessory proteins, such as CpxP, often play an important role in modulating a two-component system. Here, we investigated for the first time the parameters of interaction between a full-length reconstituted sensor histidine kinase and its cognate response regulator as well as its accessory protein. To this end, we have chosen nanodisc (ND) membranes as tool for analyses of the biochemical activities of a bacterial sensor histidine kinase and its affinities for functional interactors. The key advantage of NDs is that proteins embedded in them are accessible from both sides of the membrane bilayer. The physicochemical properties of the sensor histidine kinase CpxA incorporated into ND (CpxA-ND) indicate that the bulk of CpxA is successfully reconstituted and functional, whereas aggregated CpxA and empty NDs can be removed via SEC. CpxA is reconstituted as two dimers per ND, or as one dimer per ND to a nearly equal extent, using a 1.5-fold excess of MSP1D1. This observation is compatible with previous results [30] suggesting that the cytosolic domain of CpxA, which consists of the HAMP, DHp and CA domains, exists as a mixture of dimers and tetramers in solution. TEM analysis demonstrated successful formation of CpxA-NDs without significant concomitant creation of PLS as a byproduct. Additionally, the diameter and the shape of the CpxA-NDs were in accordance with the expected values [32, 43]. Moreover, the autophosphorylation activities of CpxA-ND and CpxA-PLS were comparable and the phosphotransfer activity was significantly higher for CpxA-ND.

The nanodisc approach allowed us to determine the affinity of the full-length, membrane-embedded histidine kinase CpxA for its cognate response regulator CpxR. Importantly, the affinity between a sensor histidine kinase and its response regulator demonstrates specificity that is displayed by kinetic preference for phosphotransfer from the former to the latter [4]. However, affinities to their cognate response regulators were so far either determined for soluble cytosolic histidine kinases such as TcrY, CrdS or CheY [4, 9] or for truncated soluble catalytic domains of sensor histidine kinases such as for EnvZ, AgrC or AHK [7, 8, 10, 11]. We assayed the affinities between CpxA-ND and CpxR by SPR in combination with IM^®^ analysis and found that an approximately 10-fold increase in binding strength between CpxA-ND and CpxR is observed in the presence of ATP. In addition to SPR, we used microscale thermophoresis (MST) to verify these results. In contrast to SPR, which is based on surface immobilization, the MST technique works in solution and employs loaded capillaries to investigate the motion of binding partners induced by a temperature gradient [49, 50]. Although the absolute values obtained with MST were higher than those from the SPR and IM approach, the 10-fold increase in binding strength between CpxA-ND and its response regulator CpxR seen in the presence of ATP was confirmed. The difference in the absolute values might be due to the fact that affinities are measured at steady-state by MST, unlike SPR, in which affinities are derived from the association and dissociation rates. Another possible explanation for this difference is that fluorescence labeling of CpxA might affect the binding affinity of CpxA for CpxR. The tenfold rise in the affinity of CpxA-ND for CpxR in the presence of ATP agrees with recent findings for the AgrAC quorum-sensing TCS of *Staphylococcus aureus* [11], in which ATP doubles the affinity of the cytosolic domain of the sensor kinase AgrC (AgrC_Cyto_) for the response regulator AgrA.

According to our results the sensor histidine kinase CpxA and the response regulator CpxR form a stable complex. This complex formation is conceivable since in contrast to most other TCSs, a considerable high proportion of CpxR is phosphorylated by acetyl-phosphate in a CpxA-independent manner [48]. Thereby, CpxR receives stimuli that are associated with growth and central metabolism and depend on the Pta-AckA pathway, and are independent of CpxA activation. Moreover, we observed a higher dissociation rate of the two proteins under non-activating conditions, perhaps indicating that the phosphatase function of CpxA is important to modulate the cellular levels of phosphorylated CpxR. Hence, the default mode of action of CpxA might be as a phosphatase. In this context, parallels with the LiaRS system become obvious, as it has been suggested that in LiaS the phosphatase activity predominates [51].

Importantly, incorporation of CpxA into nanodiscs permitted determination of the affinity between a sensor histidine kinase and its accessory protein. Since SPR is not suitable for detection of low-affinity interactions due to unspecific interactions of proteins with the dextran matrix of the sensor surface at high concentrations, we sought to investigate the interaction between sensor histdine kinase CpxA and its accessory protein CpxP via MST. However, the point of saturation for the interaction between CpxA-ND and CpxP could not be reached, revealing an apparent K_D_ > 100 μM. However, all MST measurements were conducted in aqueous solution, which does not take the impact of macromolecular crowding into account. Macromolecular crowding affects the rate constants and equilibrium constants of protein-protein interactions. Accordingly, dissociation constants are altered by favoring association kinetics [52]. In addition, the diffusion coefficient of proteins in the periplasmic space is reduced by up to 40-fold relative to the diffusion coefficient in buffer [53, 54]. The periplasmic space comprises about 20% of the bacterial cell volume [55], and this space limitation might further increase the effective protein concentrations. This leads to a drastic reduction in diffusion in the periplasmic space, which is presumed to be caused predominantly by macromolecular crowding which is characterized by increased microviscosity, transient binding, hydrodynamic effects and confinement [54]. Hence, macromolecular crowding can enhance interactions between proteins present in low copy numbers under wild-type conditions [56]. This is in line with observations that the periplasmic accessory protein CpxP measurably inhibits the Cpx response only when *cpxP* is overexpressed [34, 35, 37]. From this we conclude that the molar ratio of CpxA to CpxP is critical to render CpxP capable of inhibiting CpxA autokinase activity.

In summary, our kinetic analysis of the interaction between a sensor histidine kinase and its interacting regulatory protein offers new insights into two-component signaling. Accordingly, we postulate that the primary role of CpxP is to act as a chaperone for misfolded proteins rather than as an inhibitor of CpxA, and that CpxA and CpxR form a stable complex with a prolonged half-life upon signaling.

## Supporting Information

S1 Fig Phosphorylation of CpxR by SEC samples. To verify functional reconstitution of CpxA in ND transphosphorylation of CpxR by SEC samples was analyzed. Phosphotransfer was initiated by concomitant addition of CpxR and ATP. Samples were withdrawn after 15 min and subjected to a Zn^2+^-Phos-tag^™^ PAGE. Numbering corresponds to peak labeling in Fig 2A. As loading controls purified CpxA, purified CpxR, purified MSP and acetyl-phosphate phosphorylated CpxR were used.
(TIF)

## References

[1] Stock AM, Robinson VL, Goudreau PN. Two-component signal transduction. Annu. Rev. Biochem. 2000;69:183–215. 10966457 10.1146/annurev.biochem.69.1.183

[2] Szurmant H, Hoch JA. Interaction fidelity in two-component signaling. Curr. Opin. Microbiol. 2010;13(2):190–7. 10.1016/j.mib.2010.01.007 20133181 PMC2847666

[3] Laub MT, Goulian M. Specificity in two-component signal transduction pathways. Annu. Rev. Genet. 2007;41:121–45. 18076326 10.1146/annurev.genet.41.042007.170548

[4] Willett JW, Tiwari N, Muller S, Hummels KR, Houtman JC, Fuentes EJ, et al Specificity residues determine binding affinity for two-component signal transduction systems. Mbio. 2013;4(6):e00420–13. 10.1128/mBio.00420-13 24194534 PMC3892784

[5] Casino P, Rubio V, Marina A. Structural insight into partner specificity and phosphoryl transfer in two-component signal transduction. Cell. 2009;139(2):325–36. 10.1016/j.cell.2009.08.032 19800110

[6] Podgornaia AI, Laub MT. Determinants of specificity in two-component signal transduction. Curr. Opin. Microbiol. 2013;16(2):156–62. 10.1016/j.mib.2013.01.004 23352354

[7] Mattison K, Kenney LJ. Phosphorylation alters the interaction of the response regulator OmpR with its sensor kinase EnvZ. J. Biol. Chem. 2002;277(13):11143–8. 11799122 10.1074/jbc.M111128200

[8] Yoshida T, Cai SJ, Inouye M. Interaction of EnvZ, a sensory histidine kinase, with phosphorylated OmpR, the cognate response regulator. Mol. Microbiol. 2002;46(5):1283–94. 12453215 10.1046/j.1365-2958.2002.03240.x

[9] Bhattacharya M, Biswas A, Das AK. Interaction analysis of TcrX/Y two component system from *Mycobacterium tuberculosis*. Biochimie. 2010;92(3):263–72. 10.1016/j.biochi.2009.11.009 19962420

[10] Bauer J, Reiss K, Veerabagu M, Heunemann M, Harter K, Stehle T. Structure-Function Analysis of *Arabidopsis thaliana* Histidine Kinase AHK5 Bound to Its Cognate Phosphotransfer Protein AHP1. Mol. Plant. 2013;6(3):959–70. 10.1093/mp/sss126 23132142

[11] Srivastava SK, Rajasree K, Fasim A, Arakere G, Gopal B. Influence of the AgrC-AgrA Complex on the Response Time of *Staphylococcus aureus* Quorum Sensing. J. Bacteriol. 2014;196(15):2876–88. 10.1128/JB.01530-14 24858185 PMC4135676

[12] Buelow DR, Raivio TL. Three (and more) component regulatory systems—auxiliary regulators of bacterial histidine kinases. Mol. Microbiol. 2010;75(3):547–66. 10.1111/j.1365-2958.2009.06982.x 19943903

[13] Krell T, Lacal J, Busch A, Silva-Jimenez H, Guazzaroni ME, Ramos JL. Bacterial Sensor Kinases: Diversity in the Recognition of Environmental Signals. Annu. Rev. Microbiol. 2010;64:539–59. 10.1146/annurev.micro.112408.134054 20825354

[14] Jung K, Fried L, Behr S, Heermann R. Histidine kinases and response regulators in networks. Curr. Opin. Microbiol. 2012;15(2):118–24. 10.1016/j.mib.2011.11.009 22172627

[15] Silhavy TJ, Kahne D, Walker S. The bacterial cell envelope. Cold. Spring Harb. Perspect. Biol. 2010;2(5):a000414 10.1101/cshperspect.a000414 20452953 PMC2857177

[16] Heermann R, Jung K. The complexity of the 'simple' two-component system KdpD/KdpE in *Escherichia coli*. FEMS Microbiol. Lett. 2010;304(2):97–106. 10.1111/j.1574-6968.2010.01906.x 20146748

[17] Fleischer R, Heermann R, Jung K, Hunke S. Purification, reconstitution, and characterization of the CpxRAP envelope stress system of *Escherichia coli*. J. Biol. Chem. 2007;282(12):8583–93. 17259177 10.1074/jbc.M605785200

[18] Bernal-Cabas M, Ayala JA, Raivio TL. The Cpx Envelope Stress Response Modifies Peptidoglycan Cross-Linking via the L,D-Transpeptidase LdtD and the Novel Protein YgaU. J. Bacteriol. 2015;197(3):603–14. 10.1128/JB.02449-14 25422305 PMC4285979

[19] Bury-Mone S, Nomane Y, Reymond N, Barbet R, Jacquet E, Imbeaud S, et al Global Analysis of Extracytoplasmic Stress Signaling in *Escherichia coli*. Plos Genet. 2009;5(9): e1000651 10.1371/journal.pgen.1000651 19763168 PMC2731931

[20] Raivio TL. Everything old is new again: An update on current research on the Cpx envelope stress response. Bba-Mol. Cell Res. 2014;1843(8):1529–41. 10.1016/j.bbamcr.2013.10.018 24184210

[21] Vogt SL, Evans AD, Guest RL, Raivio TL. The Cpx Envelope Stress Response Regulates and Is Regulated by Small Noncoding RNAs. J. Bacteriol. 2014;196(24):4229–38. 10.1128/JB.02138-14 25246476 PMC4248847

[22] Dorel C, Lejeune P, Rodrigue A. The Cpx system of *Escherichia coli*, a strategic signaling pathway for confronting adverse conditions and for settling biofilm communities? Res. Microbiol. 2006;157(4):306–14. 16487683 10.1016/j.resmic.2005.12.003

[23] Rowley G, Spector M, Kormanec J, Roberts M. Pushing the envelope: extracytoplasmic stress responses in bacterial pathogens. Nat. Rev. Microbiol. 2006;4(5):383–94. 16715050 10.1038/nrmicro1394

[24] Raivio TL. Envelope stress responses and Gram-negative bacterial pathogenesis. Mol. Microbiol. 2005;56(5):1119–28. 15882407 10.1111/j.1365-2958.2005.04625.x

[25] Marman HE, Mey AR, Payne SM. Elongation factor P and modifying enzyme PoxA are necessary for virulence of *Shigella flexneri*. Infect. Immun. 2014;82(9):3612–21. 10.1128/IAI.01532-13 24935977 PMC4187845

[26] Nakayama SI, Watanabe H. Identification of cpxR as a positive regulator essential for expression of the *Shigella sonnei* virF gene. J. Bacteriol. 1998;180(14):3522–8. 9657992 10.1128/jb.180.14.3522-3528.1998 PMC107317

[27] Hung DL, Raivio TL, Jones CH, Silhavy TJ, Hultgren SJ. Cpx signaling pathway monitors biogenesis and affects assembly and expression of P pili. EMBO J. 2001;20(7):1508–18. 11285215 10.1093/emboj/20.7.1508 PMC145513

[28] MacRitchie DM, Buelow DR, Price NL, Raivio TL. Two-component signaling and gram negative envelope stress response systems. Adv. Exp. Med. Biol. 2008;631:80–110. 10.1007/978-0-387-78885-2_6 18792683

[29] Hunke S, Keller R, Muller VS. Signal integration by the Cpx-envelope stress system. FEMS Microbiol. Lett. 2012;326(1):12–22. 10.1111/j.1574-6968.2011.02436.x 22092888

[30] Mechaly AE, Sassoon N, Betton JM, Alzari PM. Segmental Helical Motions and Dynamical Asymmetry Modulate Histidine Kinase Autophosphorylation. PLoS biology. 2014;12(1). e1001776 10.1371/journal.pbio.1001776 24492262 PMC3904827

[31] Zhou X, Keller R, Volkmer R, Krauss N, Scheerer P, Hunke S. Structural basis for two-component system inhibition and pilus sensing by the auxiliary CpxP protein. J. Biol. Chem. 2011;286(11):9805–14. 10.1074/jbc.M110.194092 21239493 PMC3059015

[32] Denisov IG, Grinkova YV, Lazarides AA, Sligar SG. Directed self-assembly of monodisperse phospholipid bilayer Nanodiscs with controlled size. J. Am. Chem. Soc. 2004;126(11):3477–87. 15025475 10.1021/ja0393574

[33] Kwon E, Kim DY, Ngo TD, Gross CA, Gross JD, Kim KK. The crystal structure of the periplasmic domain of *Vibrio parahaemolyticus* CpxA. Protein Sci. 2012;21(9):1334–43. 10.1002/pro.2120 22760860 PMC3631362

[34] Raivio TL, Popkin DL, Silhavy TJ. The Cpx envelope stress response is controlled by amplification and feedback inhibition. J. Bacteriol. 1999;181(17):5263–72. 10464196 10.1128/jb.181.17.5263-5272.1999 PMC94031

[35] Danese PN, Silhavy TJ. CpxP, a stress-combative member of the Cpx regulon. J. Bacteriol. 1998;180(4):831–9. 9473036 10.1128/jb.180.4.831-839.1998 PMC106961

[36] Isaac DD, Pinkner JS, Hultgren SJ, Silhavy TJ. The extracytoplasmic adaptor protein CpxP is degraded with substrate by DegP. Proc. Natl. Acad. Sci. 2005;102(49):17775–9. 16303867 10.1073/pnas.0508936102 PMC1308919

[37] Tschauner K, Hörnschemeyer P, Müller VS, Hunke S. Dynamic Interaction between the CpxA Sensor Kinase and the Periplasmic Accessory Protein CpxP Mediates Signal Recognition in *E*. *coli*. PloS one. 2014;9(9). ARTN e107383 10.1371/journal.pone.0107383 PMC4160245 25207645

[38] Bayburt TH, Sligar SG. Membrane protein assembly into Nanodiscs. FEBS Lett. 2010;584(9):1721–7. 10.1016/j.febslet.2009.10.024 19836392 PMC4758813

[39] DeGrip WJ, VanOostrum J, Bovee-Geurts PHM. Selective detergent-extraction from mixed detergent/lipid/protein micelles, using cyclodextrin inclusion compounds: a novel generic approach for the preparation of proteoliposomes. Biochem J. 1998;330:667–74. 9480873 10.1042/bj3300667 PMC1219188

[40] Laemmli UK. Cleavage of Structural Proteins during Assembly of Head of Bacteriophage-T4. Nature. 1970;227(5259):680 5432063 10.1038/227680a0

[41] Kinoshita E, Kinoshita-Kikuta E. Improved Phos-tag SDS-PAGE under neutral pH conditions for advanced protein phosphorylation profiling. Proteomics. 2011;11(2):319–23. 10.1002/pmic.201000472 21204258

[42] Altschuh D, Bjorkelund H, Strandgard J, Choulier L, Malmqvist M, Andersson K. Deciphering complex protein interaction kinetics using Interaction Map. Biochem. Biophys. Res. Commun. 2012;428(1):74–9. 10.1016/j.bbrc.2012.10.008 23063847

[43] Shi L, Shen QT, Kiel A, Wang J, Wang HW, Melia TJ, et al SNARE proteins: one to fuse and three to keep the nascent fusion pore open. Science. 2012;335(6074):1355–9. 10.1126/science.1214984 22422984 PMC3736847

[44] Gobin AS, Rhea R, Newman RA, Mathur AB. Silk-fibroin-coated liposomes for long-term and targeted drug delivery. Int. J. Nanomed. 2006;1(1):81–7. 10.2147/nano.2006.1.1.81 PMC2426758 17722265

[45] Iuchi S. Phosphorylation Dephosphorylation of the Receiver Module at the Conserved Aspartate Residue Controls Transphosphorylation Activity of Histidine Kinase in Sensor Protein Arcb of *Escherichia Coli*. J. Biol. Chem. 1993;268(32):23972–80. 8226939

[46] Barbieri CM, Stock AM. Universally applicable methods for monitoring response regulator aspartate phosphorylation both *in vitro* and *in vivo* using Phos-tag-based reagents. Anal. Biochem. 2008;376(1):73–82. 10.1016/j.ab.2008.02.004 18328252 PMC2504525

[47] McCleary WR, Stock JB. Acetyl phosphate and the activation of two-component response regulators. J. Biol. Chem. 1994;269(50):31567–72. 7989325

[48] Wolfe AJ, Parikh N, Lima BP, Zemaitaitis B. Signal integration by the two-component signal transduction response regulator CpxR. J. Bacteriol. 2008;190(7):2314–22. 10.1128/JB.01906-07 18223085 PMC2293188

[49] Wienken CJ, Baaske P, Rothbauer U, Braun D, Duhr S. Protein-binding assays in biological liquids using microscale thermophoresis. Nat. Commun. 2010;1. ARTN 100 10.1038/ncomms1093 20981028

[50] Jerabek-Willemsen M, Wienken CJ, Braun D, Baaske P, Duhr S. Molecular Interaction Studies Using Microscale Thermophoresis. Assay Drug Dev. Techn. 2011;9(4):342–53. 10.1089/adt.2011.0380 PMC3148787 21812660

[51] Schrecke K, Jordan S, Mascher T. Stoichiometry and perturbation studies of the LiaFSR system of *Bacillus subtilis*. Mol. Microbiol. 2013;87(4):769–88. 10.1111/mmi.12130 23279150

[52] Minton AP. The influence of macromolecular crowding and macromolecular confinement on biochemical reactions in physiological media. J. Biol. Chem. 2001;276(14):10577–80. 11279227 10.1074/jbc.R100005200

[53] Mullineaux CW, Nenninger A, Ray N, Robinson C. Diffusion of green fluorescent protein in three cell environments in *Escherichia coli*. J. Bacteriol. 2006;188(10):3442–8. 16672597 10.1128/JB.188.10.3442-3448.2006 PMC1482841

[54] Sochacki KA, Shkel IA, Record MT, Weisshaar JC. Protein diffusion in the periplasm of *E*. *coli* under osmotic stress. Biophys. J. 2011;100(1):22–31. 10.1016/j.bpj.2010.11.044 21190653 PMC3010016

[55] Stock JB, Rauch B, Roseman S. Periplasmic space in *Salmonella typhimurium* and *Escherichia coli*. J. Biol. Chem. 1977;252(21):7850–61. 334768

[56] Martin J, Hartl FU. The effect of macromolecular crowding on chaperonin-mediated protein folding. Proc. Natl. Acad. Sci. U. S. A. 1997;94(4):1107–12. 9037014 10.1073/pnas.94.4.1107 PMC19752

