# Supplementary figures and images for: Interaction Analysis of a Two-Component System Using Nanodiscs
Source: PLoS One. 2016 Feb 16;11(2):e0149187. doi: 10.1371/journal.pone.0149187 (PMC4755656; doi:10.1371/journal.pone.0149187)

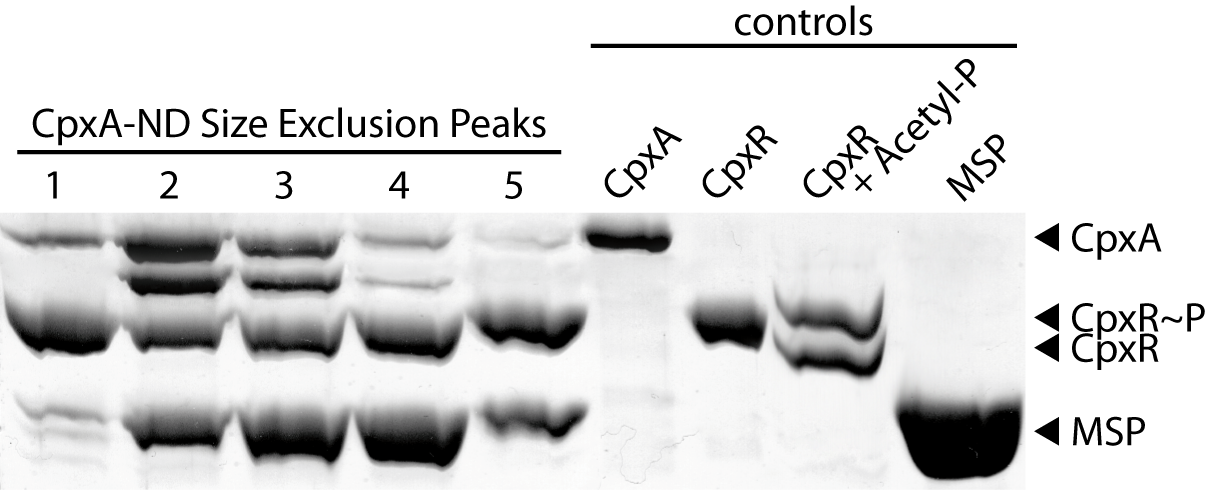

Supplement: S1 Fig — To verify functional reconstitution of CpxA in ND transphosphorylation of CpxR by SEC samples was analyzed. Phosphotransfer was initiated by concomitant addition of CpxR and ATP. Samples were withdrawn after 15 min and subjected to a Zn2+-Phos-tag™ PAGE. Numbering corresponds to peak labeling in Fig 2A. As loading controls purified CpxA, purified CpxR, purified MSP and acetyl-phosphate phosphorylated CpxR were used. (TIF) [file pone.0149187.s001.tif]
